# A Novel Construct to Treat Destructive Osteomyelitis of the Lumbar Spine in a Patient With Pre-existing Paraplegia

**DOI:** 10.7759/cureus.25162

**Published:** 2022-05-20

**Authors:** Karen Eliahu, Gregory W Basil, Michael Y Wang

**Affiliations:** 1 Neurological Surgery, University of Miami Miller School of Medicine, Miami, USA

**Keywords:** anterior column stabilization, corpectomy in paraplegic patient, thecal sac sacrifice, posterior approach, vertebral osteomyelitis

## Abstract

Treatment for vertebral osteomyelitis varies depending on the extent of pathology and includes both medical and surgical approaches. Pathogen-directed antibiotic therapy is often the first-line treatment, however, refractory cases or those with sepsis, segmental instability, or epidural abscess may be candidates for surgical treatment. Patients with extensive bony destruction often require a corpectomy with the placement of a cage for anterior column reconstruction. In this case report, we describe a patient with a complex past medical history, including paraplegia secondary to a spinal cord infarct, chronic urinary tract infections (UTIs), acute myeloid leukemia (AML), and decubitus ulcers who presented with increasing back pain and imaging demonstrating vertebral osteomyelitis and diskitis with associated epidural abscess extending from L1-L4 vertebral bodies and significant osseous destruction of the L3 and L5 vertebral bodies. A multistage surgical approach was performed involving an initial laminectomy, wound wash-out, and bony debridement followed by an additional wound wash-out and then a posterior approach for corpectomy and graft placement accomplished by tying off the thecal sac. In rare cases where patients present with complete neurologic injury and extensive destructive osteomyelitis, a posterior approach for corpectomy and stabilization may be an option.

## Introduction

Vertebral osteomyelitis, also referred to as spinal osteomyelitis or spondylodiskitis, is characterized by inflammation of the osseous components of the vertebral column secondary to infection. The underlying pathologies leading to this condition are multiple and include skin ulcers and/or hematogenous dissemination from distant sites [1]. The spread of infection from the bone may result in epidural abscess formation, nerve root involvement, and destruction of the surrounding vertebrae and soft tissue [1]. Malignancy, immunosuppression, intravenous drug use, and malnutrition are risk factors for developing vertebral osteomyelitis, and Staphylococcus aureus is the most common offending pathogen [1].

Management of vertebral osteomyelitis depends upon a number of factors including the presence of sepsis, segmental instability, or epidural abscess causing neurologic compromise, which would indicate the need for surgical treatment rather than medical treatment with pathogen-associated parenteral or oral antibiotic therapy [1-3]. Surgical treatment includes debridement and spinal stabilization. Stabilization may be accomplished via pedicle screw instrumentation alone or may be supplemented by interbody grafts. In cases where a full corpectomy is required, these interbody grafts can be placed via a number of different surgical corridors, however, they are often placed via an anterior minimally invasive oblique retroperitoneal approach or minimally invasive lateral retroperitoneal approach [4-7].

In this case report, we describe a patient with a complex past medical history, including paraplegia secondary to a spinal cord infarct in 2013, chronic urinary tract infections (UTIs), acute myeloid leukemia (AML), and decubitus ulcers who presented with increasing back pain and imaging demonstrating vertebral osteomyelitis and diskitis with associated epidural abscess and osseous destruction. Given the fact that he had undergone multiple previous anterior approaches, a posterior approach was chosen, and a novel surgical technique and construct were used to debride and stabilize the patient.

## Case presentation

A 40-year-old male with a complex past medical history of paraplegia secondary to a spinal cord infarct, subsequent neurogenic bladder requiring suprapubic catheterization complicated by multiple UTIs, and a childhood history of tracheoesophageal formation requiring abdominal surgery presented with increased “back pain” and malaise. The patient had also been recently diagnosed with AML in August 2020 for which he required chemotherapy. Due to the development of sacral decubitus ulcers, chemotherapy had to be temporarily stopped, and at the time of presentation, the patient was being treated with a wound vacuum for his ulcers.

**Figure 1:**
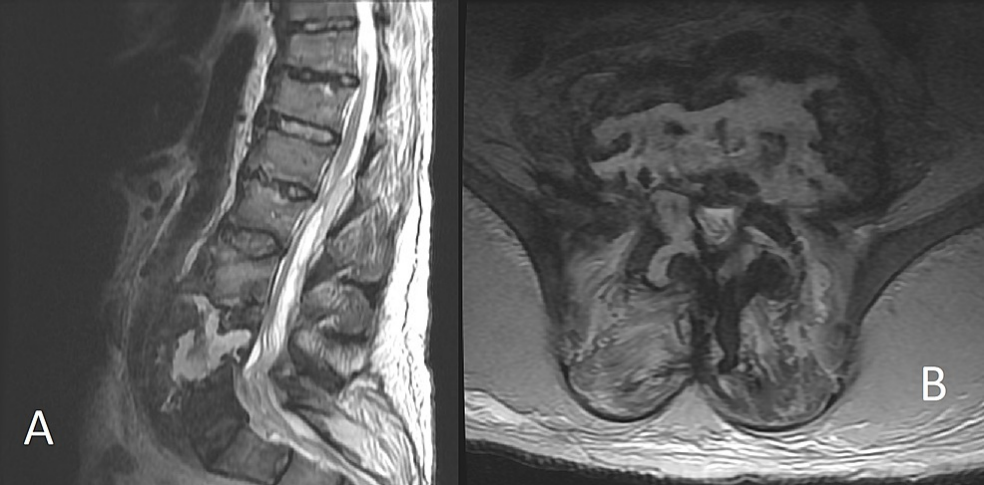
T2 sagittal (A) and axial (B) MRI of the L spine demonstrating osteomyelitis with near-complete destruction of the lumbar 4 vertebral body

Due to the presence of sacral decubitus ulcers and the clinical picture concerning for possible systemic infection, a full infectious workup was performed. At this time, a computed tomography (CT) scan of the chest and abdomen was performed and revealed significant osseous destruction of the L3 and L5 vertebral bodies. Subsequent MRI L-spine with and without contrast was performed and confirmed the diagnosis of diskitis/osteomyelitis along with a large epidural abscess extending from L1-L4 (Figure 1).

Surgical intervention

As a result of the patient’s persistent symptomatology and extensive bony destruction evidenced on imaging, the decision was made to proceed with surgical intervention. Due to the patient having undergone multiple previous abdominal surgeries, an anterior approach was not feasible. Additionally, given the active infection and evidence of large epidural collections, we opted for a multistage surgical approach to allow for partial infection resolution prior to instrumentation.

The first stage of surgery involved a laminectomy, wound washout, and bony debridement, all performed via a posterior approach. During this initial surgery, significant purulence was encountered almost immediately upon subperiosteal dissection, and following the laminectomy, the dura was noted to be densely adherent to the posterior longitudinal ligament (PLL). Small bilateral windows were created into the vertebral body lateral to the thecal sac. These windows were used to wash out and curette the superior and inferior vertebral bodies. Antibiotic-eluting beads and drains were then left in the patient.

**Figure 2:**
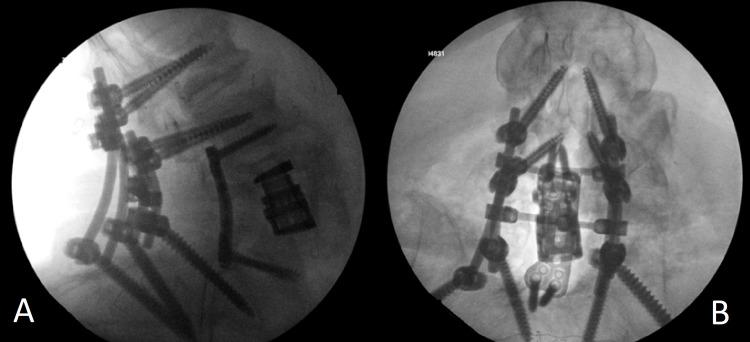
Intraoperative lateral (A) and anteroposterior (AP) (B) fluoro demonstrating construct with pedicle screw instrumentation from L2 to pelvis with an expandable interbody and four-rod construct

One week later, the patient was again brought for wash-out of the antibiotic beads, further debridement, and pulse-lavage of the paraspinal muscles. Following this intervention, the patient was maintained on an additional week of intravenous antibiotics to allow for further resolution of his infection prior to fusion. Due to the fact that the dura was densely adherent to the PLL coupled with the extensive bony destruction, the decision was made to tie off the thecal sac to allow for a posterior approach for corpectomy and fusion for the third phase of surgery. Following this maneuver, the PLL was incised and the vertebral body was entered. A large round burr was used to drill the lateral edges of the eroded vertebral bodies, and the superior and inferior endplates were prepared using curettes. Next, an expandable cage (Globus, Pennsylvania, US) was placed posteriorly and expanded to approximate the endplates. A lateral plate was then bent slightly and fixed into position using titanium screws along the remnant vertebral bodies rostral and caudal to the corpectomy. Finally, posterolateral instrumented fusion was performed, including iliac screws for added stability at the inferior aspect of the construct and a cross-link for rotational stability (Figure 2).

**Figure 3:**
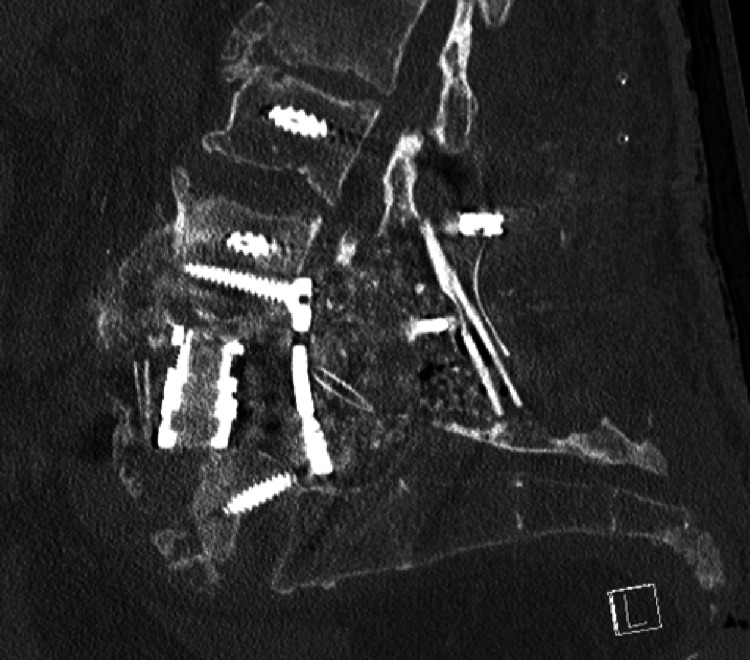
Sagittal CT of the lumbar spine demonstrating construct with an expandable cage, a lateral plate placed posteriorly, pedicle screw instrumentation, and an allograft strut placed posteriorly

A postoperative CT scan was performed and demonstrated appropriate hardware placement (Figure 3).

## Discussion

In this case report, we present a patient with a complex past medical history, who developed paraplegia secondary to a spinal cord infarct with a recent diagnosis of AML and chronic sacral decubitus ulcers. He developed vertebral osteomyelitis and diskitis following ongoing sacral pressure ulcers, which lead to an L1-L4 epidural abscess and osseous destruction of the L4 and L5 vertebral bodies.

First-line treatment for osteomyelitis and diskitis typically involves needle biopsy for pathogen identification followed by targeted antibiotic therapy [1,4]. Surgical debridement and spinal stabilization are indicated by the presence of an epidural abscess, spinal instability, poor response to antibiotic treatment, and neurologic compromise [8]. The primary goals of surgical intervention include stabilization, pain relief, improved neurologic function, and increased mobility [9]. In cases where there is extensive bony destruction, surgical intervention may include not only debridement and posterolateral stabilization but also corpectomy and placement of an interbody graft.

The anterior and lateral approaches used in corpectomy have a number of technical advantages but perhaps most importantly allow for a large corpectomy, which can then accommodate a sizable interbody graft. The benefits of these approaches are especially relevant in patients with extensive epidural extension, where scar and granulation tissue may create difficulty in dissecting the neural elements.

In the case reported within this manuscript, neither an anterior nor a lateral approach was feasible given the extensive multiple previous intra-abdominal surgeries. Additionally, given the extent of bony destruction, it was determined that a corpectomy graft would be required for adequate stability and restoration of alignment.

The surgery was planned in a staged fashion to first wash out the wound and allow for reduction of the infectious burden prior to hardware placement. Additionally, the first component of the surgery (posterior wound washout and laminectomy) would provide important information to guide the subsequent procedures, namely, whether the long-standing infection had scarred the dura to the posterior longitudinal ligament or if it was mobile and could be retracted sufficiently for graft placement. During this surgery, the dura was found to be densely adherent and inseparable from the underlying PLL, thereby necessitating tie-off of the thecal sac and performance of a posterior corpectomy as described above. The decision to tie off the thecal sac was considered a viable option in this circumstance due to the patient's previous history of paraplegia. Needless to say, this maneuver would introduce unacceptable morbidity in a patient with preserved motor function. However, there are other cases where similarly difficult pathology may be encountered in patients with spinal cord injury such as heterotopic ossification. 

The decision to place an interbody graft via a posterior approach was critical to ensure long-term stability, healing, and pain management in this patient. In cases similar to the one described in this manuscript, few alternatives exist for stabilization and fusion. Indeed, multiple previous abdominal and retroperitoneal pathologies preclude traditional minimally invasive approaches. While tying off the thecal sac should not be taken lightly, this maneuver is well-documented in the en bloc resection of sacral chordomas [10-13]. Additionally, in a patient with existing paraplegia and neurogenic bowel/bladder, the clinical impact of thecal sac sacrifice should be minimal. Conversely, the benefits of anterior column support (which would otherwise be impossible in this case) are clear and well-established in the literature [14-16]. These benefits include improved stability and sagittal plane correction [14-16]. As was the case with the patient treated in this report, the use of titanium cages for anterior column support also has significant supporting evidence [17-20].

## Conclusions

In this report, we describe a novel posterior approach, including thecal sac sacrifice, for the performance of a corpectomy and placement of an expandable cage and pedicle screw instrumentation in a paraplegic patient with osteomyelitis. This approach was considered given the extensive anterior column destruction, significant scar tissue surrounding the thecal sac, and inability to utilize an anterior or lateral approach given the previous pathology. While this technique would only be applicable in a very select patient population (namely those with complete loss of motor and sensory function), it offers a viable treatment option for the destruction of lesions of the lumbar spine where other approaches are not possible. In these populations, the benefits of anterior column reconstruction (namely, improved stability and alignment) far outweigh the minimal impact of thecal sac tie-off in patients with complete neurologic deficit.
